# Primary care physicians' perspectives towards managing rheumatoid arthritis: room for improvement

**DOI:** 10.1186/ar3517

**Published:** 2011-11-18

**Authors:** Katie L Garneau, Maura D Iversen, Hsun Tsao, Daniel H Solomon

**Affiliations:** 1 Division of Rheumatology, Brigham and Women's Hospital, 75 Francis Street, PBB-B3, Boston, MA 02115, USA; 2 Department of Physical Therapy School of Health Professions, Bouve College of Health Sciences Northeastern University, 6 Robinson Hall, Room 301C, 360 Huntington Avenue, Boston, MA 02115, USA; 3 Division of Pharmacoepidemiology, Brigham and Women's Hospital, 1620 Tremont Street, Suite 3030, Boston, MA 02120, USA

## Abstract

**Introduction:**

Many people with rheumatoid arthritis (RA) do not receive care from a rheumatologist. We surveyed primary care physicians (PCPs) to better understand their attitudes, knowledge, and practices regarding the optimal treatment of RA.

**Methods:**

Randomly selected PCPs practicing in the US were surveyed. The survey encompassed their experience with RA, use of disease modifying anti-rheumatic drugs (DMARDs), and experience with rheumatology referrals. Logistic regression analyses described the responses and examined the correlation between physician variables and use of DMARDs.

**Results:**

E-mail invitations were opened by 1, 103 PCPs and completed by 267 (25%). Most respondents were men (68%) in practice for over 10 years (64%) who reported 6 or more RA patients under their care in the last year (71%). The majority reported some RA training after medical school (59%), but only one-third felt very confident managing this condition. Most (81%) reported prescribing DMARDs, but 37% do not initiate them, with only 9% reporting being very confident starting a DMARD. In unadjusted analyses, several respondent characteristics were strongly associated with not prescribing DMARDs, but none was significant after adjustment. Almost half (44%) of PCPs noted that patients report difficulty getting appointments with rheumatologists.

**Conclusions:**

We found many PCPs are uncomfortable managing RA with DMARDs, despite common beliefs that their patients lack access to a rheumatologist. Lack of accessibility to rheumatologists and discomfort in prescribing DMARDs for patients with RA are potential barriers to optimal treatment.

## Introduction

Rheumatoid arthritis (RA) is the most common type of systemic inflammatory arthritis and causes substantial pain and disability [1]. The standard of care for RA has been well established by all professional organizations of rheumatology and includes the early initiation of disease-modifying anti-rheumatic drugs (DMARDs) [2,3]. Strong evidence supports such recommendations; early DMARD use demonstrated better long-term functional outcomes [4]. However, multiple studies from various settings show that many patients with RA do not use these drugs [5-8].

The strongest correlate of DMARD use across multiple studies is the involvement of a rheumatologist in the care of patients with RA [5,7]. Investigators have estimated that patients who see a rheumatologist are four to five times more likely to receive a DMARD than those who receive care from an internist or family practitioner [5,6]. Furthermore, at least two studies found a more favorable outcome for patients treated regularly by rheumatologists [9,10]. However, many patients with RA do not see rheumatologists.

Many factors play a role in the management of RA, but primary care physicians (PCPs) may play the biggest role since they are often the first and only point of contact for the patient. Thus, it is primarily up to the PCPs to use their own clinical knowledge to determine the 'course of action' to meet each patient's needs [11]. Studies on the management of RA have shown that, despite their abilities to diagnose RA, PCPs frequently choose not to refer patients with RA to a rheumatic disease expert; furthermore, it is rare for PCPs to initiate DMARD therapy [12,13]. Moreover, even when PCPs refer patients with RA to rheumatologists, there are too few rheumatologists to adequately care for the growing population in need of rheumatic disease expertise [9,14]. The shortage of rheumatologists manifests as long wait times for rheumatology visits or lack of rheumatology referral altogether or both [13]. Despite this evidence of a rheumatologist shortage, relatively little is known about PCPs' interactions with patients with RA [14]. Therefore, it is important to explore the factors that are related to PCPs' management of RA and that may present barriers to recommended RA care. Our aim was to study PCPs' attitudes, knowledge, and practices regarding optimal treatment of RA, including DMARD use and rheumatology referral.

## Materials and methods

### Survey participants

Participant recruitment was achieved through collaboration with a survey vendor that has access to the American Medical Association physician directory. Through this vendor, we sent e-mails to 5, 331 randomly selected physicians who had e-mail addresses in the American Medical Association physician directory and who had self-designated as 'family medicine', 'general practice', or 'internal medicine' providers. Each physician was sent the e-mail on two occasions approximately 1 month apart. The e-mail invitation included a link to the web-based survey. A $75 honorarium gift card was offered for completion of the survey. The study protocol and survey were approved by the institutional review board of Partners' Healthcare. Informed consent was implied by PCPs' completion of the survey.

### Survey

The survey consisted of sociodemographic information as well as RA-specific items. The RA-specific items focused on the respondents' experience with RA as well as their attitudes and knowledge of RA, DMARDs, and rheumatology referrals. Experience with RA was gauged through the respondents' years in practice and current number of patients with RA. Other questions ascertained physicians' attitudes and level of confidence toward referring patients with RA to rheumatologists, diagnosing RA, and prescribing DMARDs to patients with RA. Knowledge questions dealt with the appropriateness of DMARDs in RA as well as with RA training that respondents may have received beyond medical school. The survey ended with an open-ended item asking subjects whether they had any ideas for improving care for patients with RA.

### Statistical analysis

The statistical analyses described the survey responses by calculating means and frequencies. We stratified survey responses according to whether respondents reported DMARD prescribing or not. Also, survey responses were stratified on the basis of the likelihood of referral to rheumatologists. Correlates of DMARD prescribing were examined in logistic regression models that calculated the odds ratio for prescribing and 95% confidence intervals. We assessed the odds ratios for all variables and then advanced selected variables with *P *values of less than 0.10 to a multivariable logistic regression model. All analyses were conducted with SAS 9.1 (SAS Institute Inc., Cary, NC, USA).

## Results

**Figure 1:**
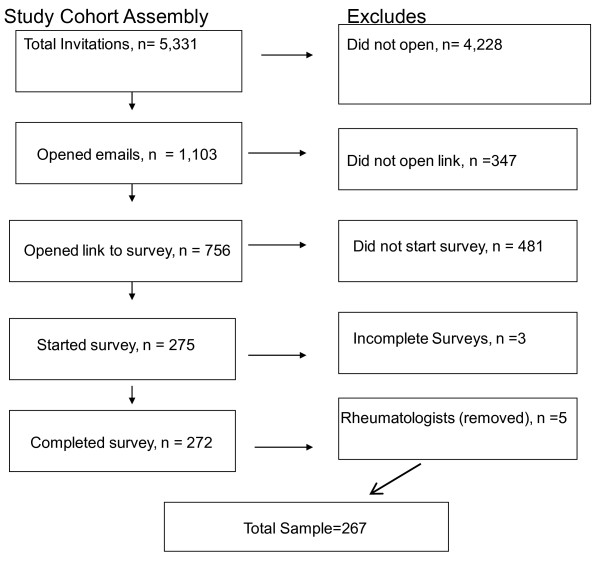
**describes the assembly of the cohort**.

Invitations were sent by e-mail to 5, 331 physicians; 1, 103 (20.7%) opened the e-mail and 756 opened the survey. Of those who opened the survey, 275 (36%) started the survey and 272 completed the survey. Five were removed from our analysis because we discovered, from their answer to an open-ended question regarding RA education beyond medical school, that they had training in rheumatology. Our total sample consisted of 267 subjects (Figure 1).

Characteristics of our sample are shown in Table 1. The majority of our sample reported being in medical practice for more than 10 years. The sample consisted of physicians representing all regions of the US. Almost two thirds of the physicians in our sample see 10 or fewer patients with RA per year. The majority of our sample stated that they were 'somewhat confident' in their ability to diagnose RA, and approximately one third described themselves as 'very confident'. Over half of the sample stated that they had received additional RA education beyond medical school; 'journals or textbooks' (39%) followed by 'residency' (37%) were the most cited educational sources. Other popular sources of RA education included 'grand rounds', 'other continuing medical education', and 'online continuing medical education'.

**Table 1:** Characteristics of survey respondents (*n *= 267)

	Number (percentage)
Female gender^a^	86 (32%)
Years practicing medicine	
0-1	4 (1%)
2-5	43 (16%)
6-10	50 (19%)
11-15	40 (15%)
16+	130 (49%)
Region of practice	
West	125 (47%)
Midwest	41 (15%)
Northeast	14 (5%)
South	86 (32%)
RA patients seen in last year	
0-5	77 (29%)
6-10	91 (34%)
11+	99 (37%)
Confidence in ability to diagnose RA	
Very confident	83 (31%)
Somewhat confident	161 (61%)
Less than confident	19 (7%)
No confidence	3 (1%)
Additional RA education beyond medical school	
Yes	156 (59%)
Source of training regarding RA	
Residency	100 (37%)
Fellowship	4 (2%)
Grand rounds	59 (22%)
Online continuing medical education	54 (20%)
Other continuing medical education	59 (22%)
Journals or textbooks or both	104 (39%)
Other conferences	48 (18%)
Other	3 (1%)

^a^Six missing. RA, rheumatoid arthritis.

We explored DMARD prescribing patterns in the survey (Table 2). Approximately one fifth of subjects stated that they did not prescribe DMARDs. Of the 217 who reported prescribing DMARDs, 45% stated that they had only continued a prescription (initially written by another physician), 9% stated that they had only initiated DMARDs, and the remainder reported writing first prescriptions and continued prescriptions. The most frequently prescribed DMARDs were non-biologics such as methotrexate, hydroxychloroquine, and sulfasalazine. However, 44% of prescribers reported prescribing biologic DMARDs.

**Table 2:** Information on prescribing disease-modifying anti-rheumatic drugs

	Total	**DMARD prescribers**^**a**^	DMARD non-prescribers
	***n *= 266**^**b**^	*n *= 217	*n *= 49
Initial prescription, continuation, or both			
First only		20 (9%)	
Continuation only		98 (45%)	
Both		99 (46%)	
Prescribed DMARDs			
Methotrexate		195 (90%)	
Hydroxychloroquine		171 (79%)	
Sulfasalazine		135 (62%)	
Leflunomide		43 (20%)	
Gold		35 (16%)	
Azathioprine		54 (25%)	
Cyclosporine		32 (15%)	
Abatacept		8 (4%)	
Etanercept		64 (29%)	
Infliximab		56 (26%)	
Rituximab		20 (9%)	
Adalimumab		43 (20%)	
Certolizumab pegol		2 (1%)	
Golimumab		1 (0%)	
Type of DMARDs prescribed^a^			
Biologic and non-biologic DMARDs		96 (44%)	
Non-biologic DMARDs only		118 (54%)	
Biologic DMARDs only		2 (1%)	
Factors that make patients inappropriate candidates for DMARDs			
No need (well controlled without DMARDs)	131 (49%)	110 (51%)	21 (43%)
Too sick to take a DMARD	92 (35%)	78 (36%)	14 (29%)
Side effects of DMARDs too problematic	148 (56%)	128 (59%)	20 (41%)
Drug interactions	83 (31%)	74 (34%)	9 (18%)
Drug cost and monitoring are too high	142 (55%)	115 (53%)	27 (55%)
Cannot get laboratory monitoring	20 (8%)	17 (8%)	3 (6%)
Best time to initiate DMARDs			
After a trial of NSAIDs or steroids	92 (35%)	75 (35%)	17 (35%)
Within the first 6 months of diagnosis	159 (60%)	132 (61%)	27 (55%)
At least 6 months after diagnosis	13 (5%)	9 (4%)	4 (8%)
Comfort level starting a DMARD^c^			
Very comfortable	23 (9%)	23 (11%)	0 (0%)
Somewhat comfortable	79 (30%)	73 (34%)	6 (12%)
Somewhat uncomfortable	112 (42%)	89 (41%)	23 (47%)
Very uncomfortable	50 (19%)	30 (14%)	20 (41%)
Comfort level continuing a DMARD^d^			
Very comfortable	75 (28%)	71 (33%)	4 (8%)
Somewhat comfortable	130 (49%)	107 (50%)	23 (47%)
Somewhat uncomfortable	54 (20%)	37 (17%)	17 (35%)
Very uncomfortable	6 (2%)	1 (0%)	5 (10%)

^a^One prescriber did not fill in types of disease-modifying anti-rheumatic drugs (DMARDs). ^b^One missing response. ^c^Three missing responses. ^d^Two missing responses. NSAID, non-steroidal anti-inflammatory drug.

When asked what the best time to initiate DMARD therapy was, a little over one half of subjects chose 'within the first 6 months of diagnosis', whereas 35% chose 'after a trial of NSAIDs [non-steroidal anti-inflammatory drugs] or steroids', and 5% chose 'at least 6 months after diagnosis'. Less than 10% of subjects reported being very comfortable initiating DMARDs. The majority (61%) of subjects stated that they were 'somewhat uncomfortable' or 'very uncomfortable' starting a DMARD; however, almost half (49%) stated that they were 'somewhat comfortable' continuing DMARD therapy. Common reasons cited for discomfort using DMARDs included 'toxicities', 'infections', and 'intravenous therapy'.

To better understand physician correlates of reporting not prescribing DMARDs, we examined the probabilities of DMARD use in logistic regression models (Table 3). In unadjusted analyses, we found several strong correlates of not prescribing DMARDs, such as fewer years in practice, fewer patients with RA, lower confidence diagnosing RA, belief that less than half of patients with RA are good candidates for DMARDs, and lacking sufficient knowledge of DMARDs. However, none of these variables was statistically significant in adjusted analyses.

**Table 3:** Probability of not prescribing a disease-modifying anti-rheumatic drug

	**Univariate**, OR (95% CI)	**Multivariate^a^**, OR (95% CI)
Years practicing medicine		
0-1	5.07 (0.69-37)	1.44 (0.14-14.83)
2-10	1.30 (0.68-2.49)	0.98 (0.48-2.01)
11+	1	1
RA patients seen in last year		
0-5	2.46 (1.30-4.67)	1.74 (0.85-3.58)
6+	1	1
Confidence in ability to diagnose RA		
Very of somewhat confident	1	1
Less than or no confidence	3.53 (1.41-8.82)	2.07 (0.72-5.95)
Additional RA education beyond medical school		
Yes	1	1
No	1.46 (0.79-2.73)	0.92 (0.45-1.89)
Proportion of RA patients who are good candidates for DMARDs		
0%-50%	2.88 (1.22-6.82)	2.41 (0.96-6.08)
51%-75%	1.01 (0.39-2.59)	0.85 (0.31-2.32)
76%-100%	1	1
Physicians' knowledge level of DMARDs		
Very knowledgeable	1	1
Somewhat knowledgeable	2.13 (0.27-16)	1.22 (0.15-10.2)
Lacking sufficient or any knowledge	9.87 (1.23-79)	5.20 (0.60-44)

^a^Multivariate model included all variables shown in the table. CI, confidence interval; DMARD, disease-modifying anti-rheumatic drug; OR, odds ratio; RA, rheumatoid arthritis.

The majority (71%) of our sample stated that they were very likely to refer patients with RA to a specialist (Table 4). When the question 'Under what situations would you refer your patients to a rheumatologist?' was asked, 'advanced disease' was the most cited reason, followed by 'patient desire' and 'uncomfortable prescribing DMARDs'. However, approximately one quarter reported that patients had difficulty getting appointments with rheumatologists. All of the physicians who were unlikely to refer gave 'no need' as their reason for not referring. However, they stated that they would refer if the patient desired. Some answers respondents gave when asked to describe 'other' reasons for referring their patients to rheumatologists included 'if they have insurance', 'for biologics or for medications requiring intravenous infusion', 'if not responding to DMARDs or failing NSAIDs', and 'compliance'. Some of the main reasons the PCPs in our sample did not refer included 'insurance problems', 'too difficult to get a rheumatology appointment', and 'no need'. Seven percent of the total sample stated their reason for not referring as 'do not know a practicing rheumatologist'.

**Table 4:** Factors influencing referral to rheumatologist for rheumatoid arthritis

		Likelihood of referring		
	Total cohort	Very likely	Possible	Unlikely
	(*n *= 266^a^)	(*n *= 189)	(*n *= 73)	(*n *= 4)
Situations leading up to referral				
Advanced disease	213 (80%)	153 (81%)	60 (82%)	0 (0%)
Patient desire for a referral	211 (79%)	153 (81%)	54 (74%)	4 (100%)
Uncomfortable prescribing DMARDs	186 (70%)	152 (80%)	33 (45%)	1 (25%)
Patient comorbidities	107 (40%)	85 (45%)	20 (27%)	2 (50%)
Other	14 (5%)	11 (6%)	3 (4%)	0 (0%)
Patients report of difficulty getting rheumatology appointment^b^				
Yes, most of the time	74 (44%)	49 (40%)	25 (57%)	0 (0%)
No, never	95 (56%)	74 (60%)	19 (43%)	2 (50%)
Main reason for not referring				
Do not know a rheumatologist	19 (7%)	10 (5%)	9 (12%)	0 (0%)
Rheumatology appointment too difficult to get	73 (27%)	46 (24%)	27 (37%)	0 (0%)
No need	63 (24%)	32 (17%)	27 (37%)	4 (100%)
Insurance problems	107 (40%)	74 (39%)	33 (45%)	0 (0%)
Other	48 (18%)	37 (20%)	11 (15%)	0 (0%)

^a^One missing response. ^b^One hundred three missing responses because of an error in the electronic survey. DMARD, disease-modifying anti-rheumatic drug.

## Discussion

We studied the prescribing and referring habits of a national sample of PCPs who took part in an e-mailed survey. Most of our subjects had more than 10 years of practice and saw six or more patients with RA in the last year. Nearly all subjects felt either somewhat confident or very confident in their ability to diagnose RA, and most had received additional RA education beyond medical school. Approximately one fifth of respondents reported not prescribing DMARDs, and although 46% of those who did claim to prescribe DMARDs stated that they would both initiate and continue DMARD therapy, 45% stated that they would only continue DMARD therapy and 9% stated they would only initiate DMARDs. Of the 81% of our sample who reported prescribing a DMARD, just over half stated that they were either 'somewhat' or 'very' uncomfortable starting a DMARD. Most of our sample stated that they would refer their patients with RA to rheumatologists, but 44% noted that patients report difficulty getting appointments with a rheumatologist.

These results suggest that PCPs commonly continue DMARDs but that a minority initiate them. A high level of discomfort prescribing DMARDs with difficulty accessing rheumatology referrals may underpin reports of suboptimal prescribing of DMARDs. There is also evidence of early use of DMARDs; however, 40% of those PCPs who prescribe DMARDs report that delayed initiation is appropriate (Table 2). This 'wait and see' approach implies a lack of urgency toward aggressive treatment and may contribute to the 'no need' attitude, felt by some, for referral to a rheumatologist (Table 4).

Furthermore, when asked what factors make patients inappropriate candidates for DMARD therapy, approximately half of the total respondents reported that there was no need for a DMARD, one third noted patients were too sick to get a DMARD, and more than half felt that side effects of DMARDs were too problematic (Table 2). Other concerns raised were drug interactions and the cost of medications and their monitoring. We asked our sample to give ideas for improving patient care in regard to RA. The most popular idea was further training and education to improve comfort level diagnosing RA and prescribing treatment. Much of our sample saw a need for more continuing medical education, and several requested a treatment algorithm to follow. Another frequently cited idea by respondents for improving RA care was increased access to rheumatologists for consultation and patient appointments as well as more patient education regarding RA and DMARD therapy.

The literature exploring PCPs' referring and prescribing habits in regard to RA management is limited. One study in Ontario, Canada, conducted a survey using a case scenario to examine PCPs' management of RA and found that most physicians would diagnose RA properly; however, rates of referral to specialists tended to be low [13]. Another Canadian study surveyed family physicians in Quebec and found that the vast majority of physicians would make proper RA diagnoses in a vignette case and that about three fourths said that they would refer patients to a rheumatic disease specialist; however, only 4% mentioned initiating DMARD therapy [12].

Our findings were similar to those of the aforementioned studies in regard to the ability and confidence of PCPs to make a proper diagnosis; however, the Ontario study showed that rates of referral were low whereas our sample's rate of referral was high. The Quebec study, like ours, showed that the majority of PCPs would refer patients with RA to specialists; however, only 4% mentioned initiating DMARD therapy whereas 80% of our sample stated that they would prescribe DMARDs. Our study is more recent, and this may explain the different attitudes of PCPs to the use of DMARDs. Additionally, differences may be due to different structures of the health-care systems. One additional difference that may be important to note between our study and these Canadian studies is the difference in response rates. Whereas studies by Bernatsky and colleagues [12] and Glazier and colleagues [13] yielded response rates of 31% and 68%, respectively, our study produced a response rate of only 25%. Possible reasons for this difference might be that the two Canadian studies mailed surveys via conventional mail whereas our surveys were internet-based and that the Canadian studies repeated their mailings at least two times whereas we did only one follow-up mailing. Additionally, we do not know what type of incentive the Canadian researchers offered their respondents for completing the survey and therefore these studies cannot be compared with ours.

Several limitations of this research are important to note. There are several issues regarding generalizability with internet surveys. One author [15] noted that respondents who answer internet-based surveys are those who are keyboard and internet literate - currently only a third of the adult population in the US. Second, internet based surveys frequently confuse respondents in their instructions. A third problem noted was a lack of time, leading to some respondents' preference to take the survey in locations other than online. It was suggested that respondents may then choose to print out the survey and take it in another location, and this would potentially exclude these respondents' answers, creating a bias [15]. There was also a relatively low response rate. Of those who opened the e-mail, 25% completed the survey. This response rate, while lower than one might hope, is similar to that of other e-mailed surveys [16]. Additionally, literature supporting the reliability of internet questionnaires as a surveying tool exists [17]. Our survey was not a validated questionnaire but a compilation of questions that regarded attitudes toward and knowledge about RA and that deserved further attention. Finally, we were unable to determine whether respondents were representative of the entire sample with regard to demographics.

## Conclusions

We surveyed a US sample of PCPs in regard to the management of RA. We found that many PCPs are uncomfortable managing RA with DMARDs, despite common beliefs that their patients lack access to a rheumatologist. Lack of accessibility to rheumatologists and discomfort in prescribing DMARDs for patients with RA are potential barriers to optimal treatment. Future directions for research into improving RA care may include the design of educational programs for PCPs to gain greater comfort prescribing DMARDs. It might also be prudent to develop ways in which rheumatic disease specialists can work more closely with PCPs to treat RA by using a 'team' approach. Such models have been developed for diabetes and depression [18,19]. Other options for improving treatment would be to further involve non-physician providers, such as nurse practitioners or physician assistants, in managing DMARDs. If such approaches are found to be effective, RA management may be improved substantially.

## Abbreviations

DMARD: disease-modifying anti-rheumatic drug; NSAID: non-steroidal anti-inflammatory drug; PCP: primary care physician; RA: rheumatoid arthritis.

## Competing interests

DHS has received research support from Amgen (Thousand Oaks, CA, USA), Abbott (Abbott Park, IL, USA), and Lilly (Indianapolis, IN, USA) and directed an educational course funded by Bristol-Myers Squibb (Princeton, NJ, USA). He has also participated in an unpaid role in several trials funded by Pfizer Inc (New York, NY, USA) but these trials were not about rheumatoid arthritis. The other authors declare that they have no competing interests.

## Authors' contributions

KLG participated in drafting the survey, data collection, and manuscript preparation. MDI participated in drafting the survey, data analysis, and manuscript preparation. HT participated in data analysis. DHS conceived of the study and participated in design of the study, drafting of the survey, data analysis, and manuscript preparation. All authors read and approved the final manuscript.
